# CCR2 Enhances Anti-Intracellular Bacterial Infection by Modulating Macrophage Pyroptosis to Rebalance Th Immune Responses

**DOI:** 10.3390/microorganisms14061339

**Published:** 2026-06-15

**Authors:** Shuaini Yang, Jinxi Yu, Jiajia Zeng, Ruoyuan Sun, Yuqing Tuo, Lu Tan, Hong Zhang, Juan Li, Xuchun Che, Hong Bai

**Affiliations:** Key Laboratory of Immune Microenvironment and Disease of the Ministry of Education, Tianjin Institute of Immunology, Department of Immunology, Tianjin Medical University, Tianjin 300070, China; nier1998@163.com (S.Y.); yujinxi1107@tmu.edu.cn (J.Y.); zjiajia814@163.com (J.Z.); sunry0609@163.com (R.S.); tuoyuqing2021@163.com (Y.T.); tanlu@tmu.edu.cn (L.T.); zhanghong0621@tmu.edu.cn (H.Z.); juanzi0223@163.com (J.L.); chexuchun@163.com (X.C.)

**Keywords:** CCR2, macrophage, pyroptosis, Th immunity, intracellular bacterial infection

## Abstract

The treatment of intracellular bacterial infections such as Chlamydia remains a significant clinical challenge due to rising antibiotic resistance and persistent, immunopathology-driven tissue damage. Macrophages are essential for host defense; they can originate from both tissue-resident precursors and circulating monocytes. During infection, macrophages at infected sites are largely derived from monocytes that migrate and differentiate there, where they phagocytose pathogens and orchestrate immune responses. The chemokine receptor CCR2 is a key regulator of this process, yet its role beyond monocyte trafficking is not fully understood. Previous studies have shown that CCR2 deficiency impairs monocyte mobilization and exacerbates disease during Chlamydia infection, shifting immune responses away from protective Th1 immunity toward pathological Th2 and Th17 polarization. Here, we investigate how CCR2 regulates macrophage function to balance protective Th1 versus pathological Th2/Th17 immunity during Chlamydia respiratory infection. Our results show that CCR2 deficiency reduces pulmonary infiltration of Ly6C^hi^ and Ly6C^low^ monocytes and shifts macrophage differentiation away from an M1-like toward an M2-like phenotype. Mechanistically, CCR2 deficiency compromises macrophage endocytosis and survival, elevates ROS production, and activates the NLRP3 inflammasome, leading to Caspase-3/GSDME-mediated pyroptosis with increased IL-1β and IL-18, while suppressing the Caspase-1/GSDMD pathway. These findings were recapitulated in vitro using *C. muridarum*-stimulated Ccr2-deficient bone marrow-derived macrophages (BMDMs), which also showed impaired migration, reduced M1-like polarization, diminished endocytosis, and enhanced ROS/NLRP3/pyroptosis. Furthermore, co-culture of these BMDMs with CD4^+^ T cells revealed that Th1 differentiation was inhibited, whereas Th2 and Th17 responses were promoted. Collectively, CCR2 orchestrates monocyte–macrophage function by driving M1-like polarization and inhibiting NLRP3/Caspase-3/GSDME pyroptosis to rebalance Th1/Th2/Th17 immunity, thereby enhancing bacterial clearance while mitigating immunopathological tissue damage during Chlamydia infection.

## 1. Introduction

Intracellular bacterial infections remain a significant clinical challenge due to rising antibiotic resistance and the lack of broadly effective vaccines. Among these pathogens, Chlamydia species are Gram-negative, obligate intracellular bacteria that cause substantial human suffering and impose a heavy socioeconomic burden worldwide. Chlamydia exhibit a characteristic biphasic developmental cycle and depend on host-derived nutrients for growth, development, and infection, having evolved sophisticated mechanisms to manipulate host metabolic and physiological processes [1]. The primary human-pathogenic species include Chlamydia trachomatis, Chlamydia psittaci, and Chlamydia pneumoniae [1]. Currently, no effective vaccine is available against chlamydial infections, and elucidating the underlying immune mechanisms is critical for developing targeted therapeutic strategies. To facilitate such investigations, we used Chlamydia muridarum (*C. muridarum*), a rodent-specific pathogen widely employed to establish murine infection models that recapitulate key features of human chlamydial disease [2,3].

Macrophages are central to host defense against intracellular pathogens, including Chlamydia. However, effective macrophage-mediated immunity requires the timely recruitment of monocytes from the bone marrow to sites of infection, a process largely governed by chemokine signaling. C-C chemokine receptor type 2 (CCR2), a key member of the chemokine receptor family, is expressed on the surface of immature dendritic cells, macrophages, monocytes, neutrophils, and activated or memory T cells [4]. Upon binding to its primary functional ligand, CCL2 [5], CCR2 initiates a G protein-mediated intracellular signaling cascade that directs monocyte migration from the bone marrow to sites of infection or inflammation [6]. CCR2 plays a pivotal role in regulating diverse biological processes, including inflammatory responses, immune surveillance, tissue repair, and organ fibrosis [7].

In mice, monocytes are primarily classified into inflammatory monocytes and patrolling monocytes. Inflammatory monocytes (CD11b^+^Ly6G^−^Ly6C^hi^) express high levels of CCR2 and low levels of CX3CR1. During inflammation, CCR2-dependent Ly6C^hi^ monocytes migrate from the bone marrow to infection sites, where they differentiate into macrophages or dendritic cells [8,9]. These cells contribute to pathogen clearance through phagocytosis, cytokine secretion, antigen presentation, and T cell activation [9]. CCR2 deficiency impairs inflammatory monocyte and tissue macrophage recruitment, increasing susceptibility to intracellular bacteria, viruses, and protozoan parasites [6]. However, in conditions such as atherosclerosis [10] and amoebic liver abscess formation [11], inflammatory monocytes may exacerbate pathology, and CCR2 deletion alleviates disease severity. Patrolling monocytes (CD11b^+^Ly6G^−^Ly6C^low^) exhibit low CCR2 but high CX3CR1 expression. These cells surveil the vascular endothelium and rapidly migrate to inflamed tissues, where they differentiate into immunoregulatory M2-type macrophages [12]. Given the critical role of CCR2 in monocyte/macrophage trafficking, we next considered the potential effector mechanisms by which these cells eliminate intracellular Chlamydia.

Pyroptosis is a Gasdermin-mediated programmed inflammatory cell death that primarily occurs in immune cells (e.g., phagocytes, macrophages, and monocytes). It is characterized by cell swelling, plasma membrane rupture, chromatin fragmentation, and release of proinflammatory molecules [13]. In infectious diseases, pyroptosis serves as a critical host defense mechanism to eliminate pathogens and respond to endogenous danger signals, while also representing a potential therapeutic target. Pyroptosis can be induced through three major pathways: the canonical, non-canonical, and a newly discovered pathway [14]. The canonical pathway involves Caspase-1 activation through NLRP3 inflammasome assembly in response to PAMPs/DAMPs, leading to GSDMD cleavage and pore formation that facilitates IL-1β/IL-18 maturation and release, thereby amplifying the inflammatory response [15]. Alternatively, the non-canonical pathway is triggered by direct LPS recognition through Caspase-4/5 (in humans) or Caspase-11 (in mice), which similarly cleaves GSDMD to induce pyroptosis [16]. Recent studies reveal that Caspase-3 can also initiate pyroptosis via GSDME cleavage, converting apoptosis into inflammatory death—a process particularly relevant in cancer immunity where GSDME activation enhances antitumor immune responses, and in immunotherapy-induced myocarditis where it exacerbates inflammatory tissue damage [17,18]. These molecular pathways collectively demonstrate the dual role of pyroptosis in host defense and immunopathology [19], highlighting its potential as a therapeutic target for infectious diseases, cancer, and inflammatory disorders. However, whether and how CCR2 signaling regulates macrophage pyroptosis to eliminate intracellular Chlamydia remains unknown.

Therefore, this study aims to determine whether CCR2 orchestrates monocyte/macrophage function by driving M1-like polarization and inhibiting NLRP3/Caspase-3/GSDME pyroptosis to rebalance Th1/Th2/Th17 immunity, thereby enhancing bacterial clearance and limiting immunopathological tissue damage during Chlamydia infection.

## 2. Materials and Methods

### 2.1. Mice

In this study, 6- to 8-week-old male C57BL/6 (wild type, WT) and Ccr2^−/−^ mice (C57BL/6 background) were used. WT mice were purchased from SiPeiFu Biotechnology (Beijing, China). Ccr2^−/−^ mice were provided by Dr. Jing Yang (Tianjin Nankai Hospital, Tianjin, China) and bred at the Tianjin Medical University Experimental Animal Center. All mice were maintained under specific-pathogen-free (SPF) conditions and handled according to the ethical guidelines for animal experiments, which were approved by the Committee on the Ethics of Animal Experiments at Tianjin Medical University (permit number: SYXK (Tianjin) 2023-0004).

### 2.2. C. muridarum Respiratory Infection Model

The respiratory infection model was established using *Chlamydia muridarum* (*C. muridarum*) as previously described. Briefly, both WT and Ccr2^−/−^ mice were anesthetized with isoflurane and then intranasally inoculated with 1 × 10^3^ inclusion-forming units (IFUs) of *C. muridarum* in 40 μL sucrose–phosphate–glutamic acid (SPG) buffer. Control mice were administered 40 μL of SPG buffer via intranasal inoculation. Tissues were harvested at 0, 3, 7, and 14 days post-infection for further analyses. All animal procedures were performed in a biosafety level 1 (BSL-1) laboratory setting.

### 2.3. Single Cells Preparation

#### 2.3.1. Bone Marrow Cells

Mice were euthanized by cervical dislocation and sterilized in 75% ethanol for 5 min. Femurs and tibias were aseptically harvested, and bone marrow was flushed with DMEM (Gibco, Thermo Fisher Scientific, Waltham, MA, USA). The cell suspension was filtered through a 70 μm strainer, centrifuged, and erythrocytes were lysed with ACK lysis buffer (Tris-NH_4_Cl). After washing, cells were resuspended in DMEM containing 10% heat-inactivated fetal bovine serum (FBS, Shanghai Life iLab Biotech, Shanghai, China) and 1% penicillin–streptomycin (Solarbio, Beijing, China).

#### 2.3.2. Peripheral Blood Leukocytes

Whole blood was collected from mice by orbital enucleation into 1.5 mL EP tubes containing 50 μL of 1% heparin sodium. After thorough mixing, the blood was transferred to a 15 mL centrifuge tube and lysed with commercial ACK lysis buffer (Solarbio, Beijing, China) according to the manufacturer’s instructions. The resulting pellet was resuspended in 1× PBS, filtered through a 70 μm cell strainer, and used for subsequent experiments.

#### 2.3.3. Lung and Spleen Single Cells

Lungs were aseptically harvested and digested with RPMI 1640 (Gibco, Thermo Fisher Scientific, Waltham, MA, USA) containing 2 mg/mL collagenase XI (Sigma-Aldrich, St. Louis, MO, USA) for 55 min at 37 °C in a 5% CO_2_ incubator. During the final 5 min of digestion, 2 mM EDTA was added. After digestion, 35% Percoll (GE Healthcare, Chicago, IL, USA) was mixed with the sample, and the mixture was centrifuged at 2000 rpm for 20 min at 12 °C to remove tissue fascia. Spleen tissue was mechanically homogenized and passed through a 70 μm cell strainer. Erythrocytes were lysed using ACK lysis buffer, and the single-cell suspensions were resuspended in RPMI-1640 supplemented with 10% FBS and 1% penicillin–streptomycin. Cell concentrations were determined by trypan blue exclusion and adjusted to 2 × 10^6^ cells/mL for lung samples and 1 × 10^7^ cells/mL for spleen samples prior to subsequent experiments.

### 2.4. Induction and Stimulation of Bone Marrow-Derived Macrophages (BMDMs)

Bone marrow cells from naïve WT and Ccr2^−/−^ mice were isolated and cultured in DMEM supplemented with 10% FBS, 1% penicillin–streptomycin, and 20 ng/mL M-CSF. The medium was replaced on days 3, 5, and 7 with fresh DMEM containing 20 ng/mL M-CSF. On day 7, mature BMDMs were divided into four treatment groups: Control (unstimulated), LPS (100 ng/mL), *C. muridarum* (MOI = 10), and MCC_950_+*C. muridarum* (10 μM MCC_950_ (S8930, Selleck Chemicals, Houston, TX, USA) pre-treatment for 1 h prior to infection). After 24 h of stimulation, cell supernatants were collected for IL-1β and IL-18 quantification by ELISA (Solarbio, Beijing, China), while cells were harvested for qPCR, Western blot, and flow cytometry.

### 2.5. Flow Cytometry Analysis

For surface staining, single cells or BMDMs were incubated with Zombie NIR Fixable Viability Kit (423106, BioLegend, San Diego, CA, USA) at room temperature for 15 min in the dark to exclude dead cells. After washing with PBS containing 2% FBS, cells were incubated with anti-CD16/CD32 (2334894, Invitrogen, Carlsbad, CA, USA, Clone: 93) for 30 min at 4 °C to block nonspecific Fc receptor binding. Cells were then stained with the antibody cocktail for 30 min at 4 °C in the dark. Anti-CD45-PerCP-Cy5.5 (103131, Clone: B440074), anti-CD11b-FITC (101206, Clone: R324794), anti-Ly6C-PE-Cy7 (2399959, Clone: HK1.4), anti-Ly6G-PE (M100L7-09D, Clone: AB627), anti-F4/80-APC (132116, Clone: B447979), anti-CD11c-FITC (117306, Clone: B370929), anti-CD80-PE (104708, Clone: B340152), anti-CD86-PE-Cy7 (105014, Clone: B360042), anti-MHC II-PE (107607, Clone: B377619), anti-CD206-PE-Cy7 (141720, Clone: B357067), and anti-CD4-APC (100411, Clone: B436831) (all from Biolegend). This staining strategy enabled identification of monocytes (CD45^+^CD11b^+^Ly6C^+^), macrophages (F4/80^+^CD11b^+^), M1, and M2 subsets.

For intracellular cytokine staining, lung single cells were stimulated with PMA (50 ng/mL, Sigma-Aldrich), ionomycine (1 μg/mL, MCE, Monmouth Junction, NJ, USA), and brefeldin A (10 μg/mL, BioLegend) for 5–6 h at 37 °C. Cells were first stained with Zombie NIR Fixable Viability Kit to exclude dead cells, followed by Fc receptor blockade with anti-CD16/32 and surface staining with anti-CD45-PerCP-Cy5.5 and anti-F4/80-APC. After fixation with Fixation Buffer (BioLegend) for 20 min at room temperature in the dark, cells were permeabilized using 1× Intracellular Staining Perm Wash Buffer (BioLegend), centrifuged at 350× *g* for 5 min, and washed twice. Intracellular staining was performed by incubating cells with anti-TNF-α-PE-Cy7 (506324, Clone: B363134), anti-IL-10-PE-Cy7 (505026, Clone: B357222), anti-TGF-β-PE (141403, Clone: B195898), anti-iNOS-PE (696806, Clone: B356090), and anti-ARG1-PE-Cy7 (2250751, Clone: AlexF5) for 20 min at room temperature in the dark. Spectral compensation was performed using single-stained cells with antibodies against CD4 or CD3 conjugated to APC-Cy7, PerCP-Cy5.5, APC, FITC, PE, and PE-Cy7. All gates were set based on fluorescence-minus-one (FMO) controls. Flow cytometry was performed on a FACS Canto II (BD Biosciences, Franklin, NJ, USA), and data were analyzed using FlowJo v10.8.1.

### 2.6. Macrophage Endocytosis Assay

Endocytic activity was assessed by measuring FITC–dextran (40 kDa, FD40S, Sigma-Aldrich) uptake via flow cytometry. BMDMs or lung single-cell suspensions (5 × 10^4^ cells) were filtered through a 70 μm mesh into flow cytometry tubes. Macrophages were identified by surface staining with anti-CD45, anti-F4/80, and anti-CD11b. After staining, cells were centrifuged at 1200 rpm for 5 min at room temperature and washed twice. After washing, cells were resuspended in RPMI 1640. The negative control group was incubated without FITC–dextran, while experimental groups were incubated with 1 mg/mL FITC–dextran at 37 °C for 30 min. All groups were then washed and resuspended in 100 μL PBS containing 2% FBS for immediate flow cytometry acquisition.

### 2.7. Cell Viability Assay

#### 2.7.1. Hoechst 33342/PI Double Staining

WT-BMDMs and Ccr2^−/−^-BMDMs were seeded on coverslips in 24-well plates at a density of 1–3 × 10^4^ cells/well and stimulated with *C. muridarum* (MOI = 10) for 24 h. Cells were sequentially stained with Hoechst 33342 and propidium iodide (PI) according to the manufacturer’s instructions. Briefly, after supernatant removal and PBS washing, cells were incubated with 0.5% Hoechst 33342 for 5 min at room temperature (RT) in the dark, washed with PBS, and then stained with 0.5% PI under the same conditions. If immediate observation was not possible, cells were fixed for 15 min at RT. Coverslips were mounted onto glass slides, sealed, and analyzed by fluorescence microscope. Hoechst 33342 (blue fluorescence) labeled all nuclei, while PI (red fluorescence) selectively stained dead cells through binding to DNA in cells with permeabilized plasma membranes.

#### 2.7.2. Annexin V/7-AAD Analysis

Annexin V/7-AAD analysis was performed using a commercial kit (CA1030, Solarbio, Beijing, China). Single-cell suspensions from lungs of *C. muridarum*-infected WT and Ccr2^−/−^ mice (day 7) were filtered through a 70 μm strainer (2.5–3 × 10^5^ cells per sample). Cells were sequentially stained with Zombie NIR Fixable Viability Dye to exclude dead cells, followed by Fc receptor blockade using anti-CD16/32. Macrophages were identified by surface staining with anti-CD45-APC-Cy7, anti-F4/80-FITC, and anti-CD11b-APC. For detection, cells were resuspended in 100 μL binding buffer containing 5 μL Annexin V-PE and 10 μL 7-AAD, incubated for 15 min (RT, dark), and diluted with 400 μL PBS. Samples were analyzed within 1 h on a BD FACS Canto II.

### 2.8. ROS Analysis

ROS production was assessed using a commercial ROS assay kit (S0033S, Beyotime, Shanghai, China) according to the manufacturer’s instructions. Briefly, BMDMs were treated as described previously. Lung single-cell suspensions (1 × 10^5^ to 2 × 10^6^ cells) from WT and Ccr2^−/−^ mice infected for 7 days were collected and filtered through a 200-mesh sieve into flow cytometry tubes. After surface staining to identify macrophages (CD45^+^F4/80^+^CD11b^+^ cells), cells were resuspended in 10 μM DCFH-DA in RPMI 1640 and incubated at 37°C with 5% CO_2_ for 20 min, with gentle mixing every 3–5 min. After washing three times with RPMI 1640 to remove excess dye, a positive control (Rosup, 1:5000 dilution) was added and incubated for 20–30 min to induce ROS production. Cells were then washed twice with PBS and analyzed by flow cytometry. The fluorescence intensity of FITC was measured to quantify ROS production.

### 2.9. LDH Release Assay

Cell viability was assessed using an LDH release kit (C0017, Beyotime, Shanghai, China). BMDMs were seeded in 96-well plates and treated as described above. Supernatants were collected, and LDH release was measured according to the manufacturer’s instructions. Absorbance was recorded at 490 nm, and LDH release was calculated as: LDH release (%) = (sample absorbance−background)/(maximum absorbance−background) × 100.

### 2.10. Scratch Assay

WT-BMDMs and Ccr2^−/−^-BMDMs were seeded in 6-well plates and allowed to mature overnight. A scratch wound was created by gently applying a sterile pipette tip perpendicular to marked lines. After aspirating the medium, cells were washed 2–3 times with sterile PBS to remove floating cells, and fresh serum-free or low-serum medium (FBS ≤ 2%) was added. Cells were then stimulated with *C. muridarum* at a multiplicity of infection (MOI) of 10. Images of the scratch area were captured immediately after stimulation (0 h) and after 24 h of incubation. Before imaging at 24 h, cells were washed with PBS to remove non-adherent cells. The scratch area was measured at both time points using Image J software(Fiji), and the cell migration rate was calculated as: (0 h scratch area−24 h scratch area)/0 h scratch area.

### 2.11. Quantitative Real-Time PCR Analysis (qPCR)

BMDMs or lung tissues were lysed with TRIzol reagent (Ambion, Austin, TX, USA) to extract total RNA according to the manufacturer’s instructions. RNA was reverse transcribed into cDNA using TransScript One-Step gDNA Removal and cDNA Synthesis SuperMix (TransGen Biotech, Beijing, China) according to the manufacturer’s instructions. qPCR was conducted on a LightCycler 96 system (Roche, Basel, Switzerland) using 2× RealStar Fast SYBR qPCR Mix (GenStar, Beijing, China). The mRNA levels of target genes were normalized to β-actin and quantified using the 2^−ΔΔCt^ method. All primers were synthesized by Shanghai Sangon Biotech, with sequences listed in Table 1.

### 2.12. Western Blot

Total protein was extracted from BMDMs or lung tissues at day 7 p.i. using RIPA lysis buffer (R0278, Millipore, Burlington, MA, USA) containing 1× protease and phosphatase inhibitor cocktail (78442, Thermo Fisher Scientific, USA). Protein samples were then mixed with loading buffer (P1040, Solarbio, Beijing, China) and heat-denatured at 100 °C in a water bath for 5–10 min to ensure complete protein denaturation. Proteins were separated by sodium dodecyl sulfate-polyacrylamide gel electrophoresis (SDS-PAGE) and transferred to polyvinylidene fluoride (PVDF) membranes. After blocking with 5% BSA in TBST for 2 h at RT, membranes were incubated overnight at 4 °C with the following primary antibodies: NLRP3 Rabbit mAb (A24294, ABclonal, Wuhan, China), Total and Cleaved Caspase-1 Antibody (M025280F, Abmart, Shanghai, China), Gasdermin D (E4M2W) Rabbit mAb (46451S, Cell Signaling Technology, Danvers, MA, USA), Caspase-3 Antibody (9662, Cell Signaling Technology, MA, USA), Cleaved Caspase-3 (Asp175) Antibody (9661T, Cell Signaling Technology, MA, USA), Gasdermin E (E1C5B) Rabbit mAb (88874S, Cell Signaling Technology, MA, USA) and β-actin Rabbit mAb (High Dilution) (A2235, ABclonal, Wuhan, China). Membranes were then incubated with Goat anti-Rabbit IgG-HRP Antibody (abs20040ss, Absin, Shanghai, China) for 1 h at room temperature. Protein bands were visualized using StarSignal High Sensitivity Chemiluminescence Kit (GenStar, Beijing, China) and imaged on Tanon-5200 Imager (Tanon, Shanghai, China).

### 2.13. BMDM and T Cell Co-Culture

To evaluate the effect of *C. muridarum*-stimulated BMDMs on T cell responses, mature BMDMs were seeded at 1 × 10^5^ cells/well in 100 μL medium in U-bottom 96-well plates and stimulated with UV-inactivated *C. muridarum* (MOI = 10) for 24 h. Splenic CD4^+^ T cells were isolated using a MACS CD4 T Cell Isolation Kit (Miltenyi Biotec, Bergisch Gladbach, Germany) according to the manufacturer’s instructions. Purified CD4^+^ T cells were co-cultured with stimulated BMDMs at a 10:1 ratio (T cells: BMDMs) for 48 h at 37 °C with 5% CO_2_. Culture supernatants were collected for cytokine analysis of IFN-γ, IL-4, and IL-17A by ELISA according to the manufacturer’s instructions (Solarbio, Beijing, China). Non-adherent T cells were harvested for RNA extraction and cDNA synthesis. Gene expression of *Tbet*, *Il4*, and *Il17a* was quantified by qPCR.

### 2.14. Statistical Analysis

All data were analyzed using GraphPad Prism 10 (GraphPad InStat Software, San Diego, CA, USA) and presented as mean ± SD. For comparisons between two groups, an unpaired Student’s *t*-test was used. For experiments involving two factors (genotype and time, or genotype and treatment), two-way ANOVA was performed, followed by Šidák’s post hoc test for multiple comparisons. Statistical significance was set at *p* < 0.05 (* *p* < 0.05, ** *p* < 0.01, *** *p* < 0.001, **** *p* < 0.0001).

## 3. Results

### 3.1. Ccr2 Deficiency Impairs Monocyte Recruitment and Promotes a Macrophage-like Phenotype During C. muridarum Respiratory Infection

**Figure 1:**
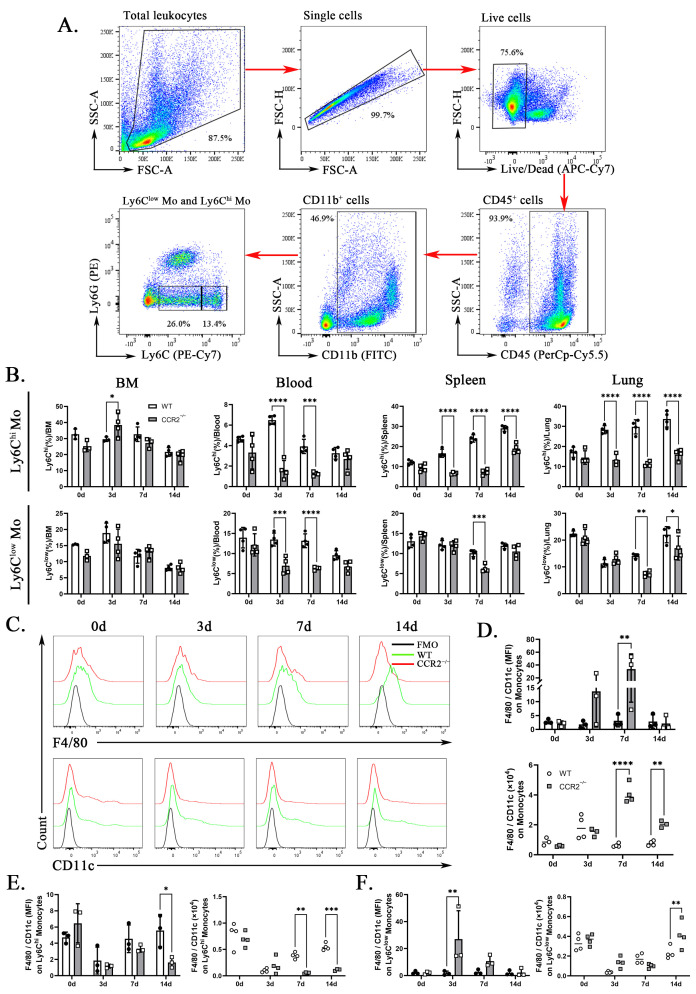
CCR2 deficiency impairs monocyte recruitment and promotes a macrophage-like phenotype during *C. muridarum* respiratory infection. (**A**) Gating strategy for lung monocyte subsets. Live, singlet CD45^+^ leukocytes were gated, followed by exclusion of Ly6G^+^ neutrophils. Ly6C^hi^ monocytes (Ly6C^hi^ Mo) were defined as CD11b^+^Ly6G^−^Ly6C^hi^ cells, and Ly6C^low^ monocytes (Ly6C^low^ Mo) as CD11b^+^Ly6G^−^Ly6C^low^ cells. (**B**) Kinetics of Ly6C^hi^ Mo (**top**) and Ly6C^low^ Mo (**bottom**) percentages in bone marrow (BM), peripheral blood, spleen, and lungs of WT and Ccr2^−/−^ mice at indicated days post-infection (p.i.) with *C. muridarum* (1 × 10^3^ IFUs, intranasal). (**C**) Representative histograms of F4/80 (macrophage marker) and CD11c (dendritic cell marker) surface expression on gated Ly6C^+^ monocytes in WT (green) and Ccr2^−/−^ (red) mice at day 7 p.i. Fluorescence-minus-one (FMO) controls are shown in black. (**D**) Ratio of F4/80 to CD11c mean fluorescence intensity (MFI, upper) and cell count ratio of F4/80^+^Ly6C^+^ to CD11c^+^Ly6C^+^ cells (lower) in total Ly6C^+^ monocytes. (**E**) F4/80/CD11c MFI ratio (**left**) and cell count ratio (**right**) in Ly6C^hi^ Mo. (**F**) F4/80/CD11c MFI ratio (**left**) and cell count ratio (**right**) in Ly6C^low^ Mo. Data are shown as mean ± SD (*n* = 3-4 mice per genotype and time point) and are representative of three independent experiments. Statistical significance was determined by two-way ANOVA. * *p* < 0.05, ** *p* < 0.01, *** *p* < 0.001, **** *p* < 0.0001.

CCR2, the primary receptor for CCL2 (MCP-1), orchestrates monocyte migration from the bone marrow (BM) into the circulation and subsequent infiltration of inflamed tissues, where CCR2^+^ monocytes differentiate into macrophages or dendritic cells to mediate antimicrobial immunity. To evaluate the impact of CCR2 deficiency on monocyte dynamics during *C. muridarum* respiratory infection, we quantified the percentages of inflammatory (Ly6C^hi^ Mo) and patrolling (Ly6C^low^ Mo) monocytes in the BM, peripheral blood, spleen, and lungs of wild-type (WT) and Ccr2^−/−^ mice at days 0, 3, 7, and 14 post-infection (p.i.) using flow cytometry. The gating strategy for identifying monocyte subsets in murine lungs is shown in Figure 1A.

In Ccr2^−/−^ mice, Ly6C^hi^ Mo accumulated markedly in the BM at day 3 p.i. compared to WT controls, whereas their proportions were significantly reduced in the peripheral blood, spleen, and lungs at days 3, 7, and 14 p.i. (Figure 1B). By contrast, the proportion of Ly6C^low^ Mo in the BM did not differ between genotypes either before or after infection. However, Ccr2^−/−^ mice exhibited consistently lower levels of Ly6C^low^ Mo in the peripheral blood, spleen, and lungs following infection (Figure 1B). Together, these data indicate that CCR2 deficiency impairs monocyte egress from the BM and their subsequent recruitment to peripheral tissues, leading to an abnormal accumulation of Ly6C^hi^ Mo in the BM.

We next examined the phenotypic changes in monocyte subsets with respect to F4/80 (a macrophage marker) and CD11c (a dendritic cell marker) expression by analyzing the mean fluorescence intensity (MFI) of F4/80 and CD11c on Ly6C^+^ Mo, Ly6C^hi^ Mo, and Ly6C^low^ Mo in lung tissues, as well as the absolute counts of F4/80^+^ and CD11c^+^ cells. As shown in Figure 1C,D, Ccr2^−/−^ mice exhibited a significantly higher MFI ratio of F4/80 to CD11c on total Ly6C^+^ Mo and an increased ratio of F4/80^+^Ly6C^+^ Mo to CD11c^+^Ly6C^+^ Mo counts at day 7 p.i., a difference that persisted until day 14 p.i. Notably, CCR2 deficiency led to a significant decrease in the F4/80/CD11c ratio in Ly6C^hi^ Mo at day 14 p.i. (Figure 1E), while concurrently elevating these ratios in Ly6C^low^ Mo at both days 7 and 14 p.i. (Figure 1F). These findings indicate changes in the balance of surface marker expression, suggesting a potential shift toward a macrophage-like phenotype in the absence of CCR2. Furthermore, the Ly6C^low^ subset might contribute to the generation of F4/80-expressing cells in the CCR2-deficient microenvironment, although direct evidence of lineage differentiation is not provided.

Collectively, these findings demonstrate that CCR2 deficiency not only compromises the recruitment of both monocyte subsets to the infected lungs but also is associated with a compensatory shift in the phenotypic profile of monocyte subsets, with an increased representation of F4/80^+^ cells among the Ly6C^low^ subset. This alternative phenotypic adaptation may help maintain pulmonary immune homeostasis during *C. muridarum* respiratory infection.

### 3.2. Ccr2 Deficiency Shifts Macrophage Polarization Toward an M2-like Phenotype and Impairs Endocytic Activity During C. muridarum Respiratory Infection

**Figure 2:**
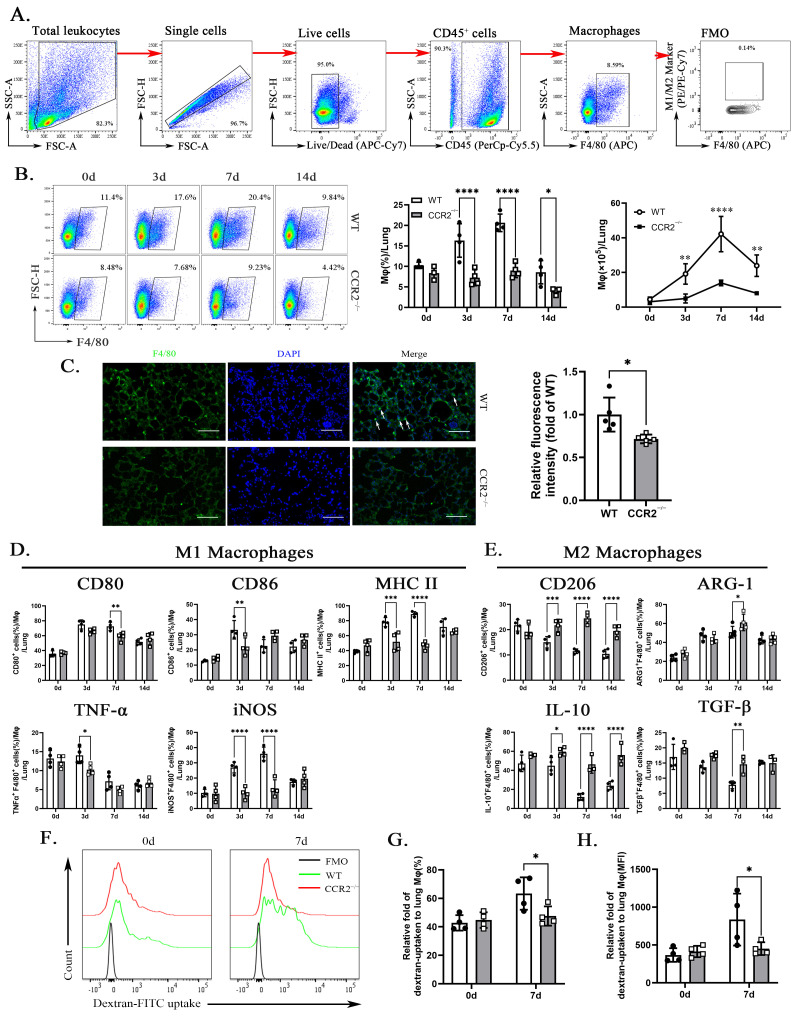
Altered macrophage polarization and impaired endocytosis in the lungs of Ccr2^−/−^ mice during *C. muridarum* respiratory infection. WT and Ccr2^−/−^ mice were intranasally inoculated with 1 × 10^3^ inclusion-forming units (IFUs) of *Chlamydia muridarum* (*C. muridarum*). Lung tissues were collected at 0, 3, 7, and 14 days p.i. and analyzed by flow cytometry. (**A**) Gating strategy for lung M1-like and M2-like macrophages based on CD45^+^F4/80^+^ cells and FMO controls. (**B**) Representative plots and quantification of the percentage and absolute number of pulmonary macrophages in WT and Ccr2^−/−^ mice. (**C**) Representative immunofluorescence staining for F4/80 (green) and DAPI (blue) at day 7 p.i. (scale bar, 100 μm, **left**) with the graph showing mean fluorescence intensity normalized to the WT group (**right**). The white arrow pointed to the representative immunofluorescence staining. (**D**) Quantitative analysis of M1-like markers in pulmonary macrophages. Percentages of CD80^+^, CD86^+^, MHC II^+^, iNOS^+^F4/80^+^, and TNF-α^+^F4/80^+^ cells in lung tissues from WT and Ccr2^−/−^ mice. (**E**) Quantitative analysis of M2-associated markers in pulmonary macrophages. Percentages of CD206^+^, ARG-1^+^F4/80^+^, IL-10^+^F4/80^+^, and TGF-β^+^F4/80^+^ cells in lung tissues from WT and Ccr2^−/−^ mice. (**F**) Representative histograms of FITC–dextran uptake at day 7 p.i. Black line: FMO control, Green line: WT mice, Red line: Ccr2^−/−^ mice. (**G**,**H**) Quantification of endocytosis as percentage of FITC^+^ cells (**G**) and MFI (**H**). Data are shown as mean ± SD (*n* = 3–4 mice per genotype and time point) and are representative of three independent experiments. Statistical significance was determined by two-way ANOVA. * *p* < 0.05, ** *p* < 0.01, *** *p* < 0.001, **** *p* < 0.0001.

Given the essential role of CCR2 in monocyte recruitment and as a precursor of tissue macrophages, we first examined whether CCR2 deficiency affects macrophage accumulation in the lung during *C. muridarum* respiratory infection. Flow cytometric analysis revealed that the proportions and absolute numbers of pulmonary macrophages were significantly lower in Ccr2^−/−^ mice than in WT controls at days 3, 7, and 14 p.i. (Figure 2B). Immunofluorescence staining confirmed a marked reduction in F4/80^+^ cell infiltration at day 7 p.i. (Figure 2C), consistent with the well-established role of CCR2 in directing macrophage trafficking to sites of inflammation [20]. We next investigated how CCR2 deficiency influences macrophage functional polarization. In Ccr2^−/−^ mice, the expression of classical M1-like markers was substantially diminished. The frequencies of CD80^+^, CD86^+^, and MHC II^+^ macrophages were all decreased (Figure 2D), accompanied by reduced production of inducible nitric oxide synthase (iNOS) at days 3 and 7 p.i. and lower TNF-α secretion at day 3 p.i. (Figure 2D). Conversely, markers associated with alternative M2-like activation were elevated in the absence of CCR2. The percentages of CD206^+^ macrophages were increased at all three time points, and ARG-1^+^ macrophages were increased at day 7 p.i. (Figure 2E). This M2-skewed phenotype was further supported by higher levels of the anti-inflammatory cytokines IL-10 (days 3–14 p.i.) and TGF-β (day 7 p.i.) (Figure 2E), suggesting that CCR2 deficiency promotes a compensatory shift toward alternative activation.

Beyond polarization, we assessed the endocytic capacity of pulmonary macrophages. At day 7 p.i., both the proportion of FITC^+^ macrophages and the MFI of FITC were significantly lower in Ccr2^−/−^ mice than in WT controls (Figure 2F–H), indicating that CCR2-deficient macrophages exhibit impaired endocytic function.

Collectively, during *C. muridarum* respiratory infection, CCR2 deficiency not only limits the overall accumulation of macrophages in the lung but also shifts their polarization from an M1-like toward an M2-like phenotype, while concurrently compromising their endocytic capacity.

### 3.3. Ccr2 Deficiency Impairs Migration, M1-like Polarization, and Endocytosis but Promotes Cell Death in C. muridarum-Stimulated BMDMs

**Figure 3:**
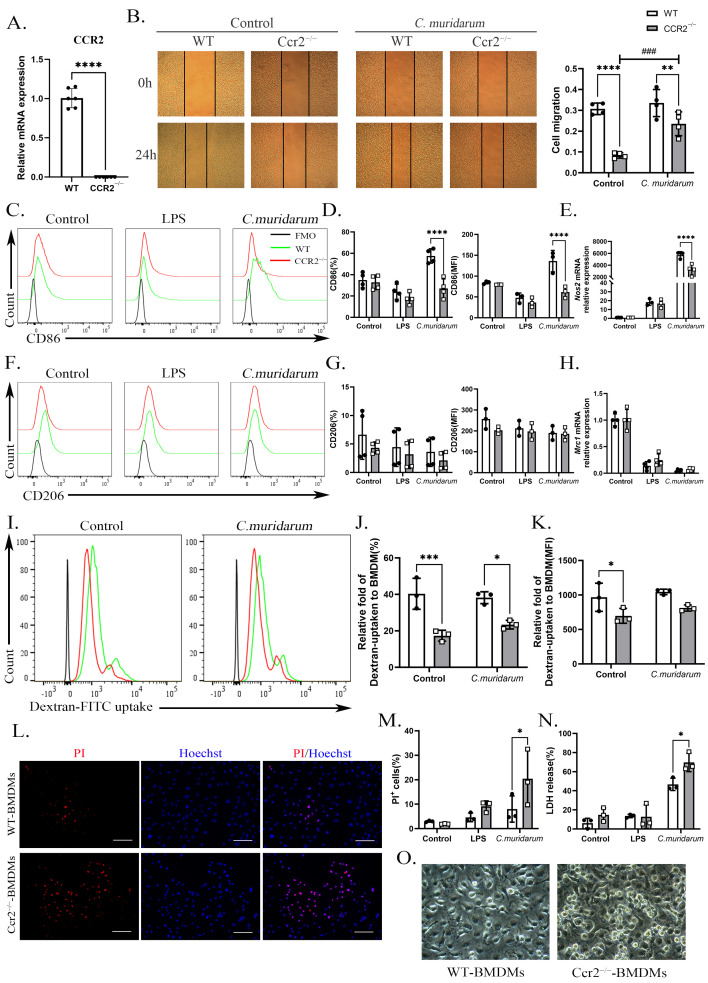
CCR2 deficiency impairs macrophage migration, M1 polarization, and survival during *C. muridarum* infection. Bone marrow cells from naïve WT and Ccr2^−/−^ mice were differentiated into bone marrow-derived macrophages (BMDMs) with M-CSF. BMDMs were left untreated or stimulated with *C. muridarum* (MOI = 10) or LPS (100 ng/mL) for 24 h. (**A**) *Ccr2* mRNA expression in BMDMs by qPCR. (**B**) Representative images from the scratch assay of WT-BMDMs and Ccr2^−/−^-BMDMs stimulated with *C. muridarum* at 0 h and 24 h are shown on the left. Statistical analysis of the cell migration rate is presented on the right. (**C**,**F**) Representative flow cytometry histograms of CD86 (**C**) and CD206 (**F**) expression. Black line: FMO, red line: Ccr2^−/−^-BMDMs, green line: WT-BMDMs. (**D**,**G**) Flow cytometry data were summarized to show the percentages (**left panel**) and MFI (**right panel**) of CD86 (**D**) and CD206 (**G**). (**E**,**H**) *Nos2* (**E**) and *Mrc1* (**H**) mRNA expression by qPCR. (**I**) Representative histograms of FITC–dextran uptake at 24 h after *C. muridarum* stimulation. (**J**) The proportion of FITC^+^ cells within BMDMs. (**K**) The MFI of FITC in BMDMs. (**L**) Morphological changes were observed with fluorescence microscopy following propidium iodide (PI)/Hoechst 33342 staining in BMDMs derived from both WT and Ccr2^−/−^ mice after a 24 h treatment with *C. muridarum*. Blue: Hoechst 33342, Red: PI. Scale bar, 100 μm. (**M**) Flow cytometry analysis of the percentage of PI-positive cells in WT-BMDMs and Ccr2^−/−^-BMDMs. (**N**) LDH release was measured in the BMDMs. (**O**) Representative images of cell morphology in WT-BMDMs (**left**) and Ccr2^−/−^-BMDMs (**right**) following 24 h of *C. muridarum* stimulation. Scale bar, 20 μm. Data are mean ± SD from three independent wells per group and are representative of three independent experiments. Statistical significance was determined by unpaired Student’s *t*-test (**A**) or two-way ANOVA. * *p* < 0.05, ** *p* < 0.01, *** *p* < 0.001, **** *p* < 0.0001, ^###^ *p* < 0.001.

Having established that CCR2 deficiency alters macrophage polarization and endocytosis in vivo, we next asked whether these defects are cell-intrinsic and recapitulated in isolated macrophages under controlled conditions. To this end, we isolated and cultured bone marrow-derived macrophages (BMDMs) from naïve WT and Ccr2^−/−^ mice (Figure 3A). Even under basal conditions, Ccr2^−/−^-BMDMs exhibited significantly reduced migration compared to WT controls. *C. muridarum* stimulation enhanced the migratory capacity of both genotypes, yet Ccr2^−/−^-BMDMs remained significantly less motile (Figure 3B), mirroring the diminished macrophage accumulation observed in infected lungs (Figure 2B). We next examined macrophage polarization. Upon *C. muridarum* stimulation, Ccr2^−/−^-BMDMs showed impaired M1-like polarization, as evidenced by a lower frequency of CD86^+^ cells, reduced CD86 MFI, and decreased *Nos2* expression (Figure 3C–E). In contrast, no significant differences were detected in the proportion of CD206^+^ cells (Figure 3F,G), CD206 MFI (Figure 3G), or *Mrc1* expression (Figure 3H), indicating that CCR2 deficiency specifically impairs proinflammatory macrophage activation without broadly affecting M2-like markers under these conditions.

Consistent with the in vivo endocytic defect, Ccr2^−/−^-BMDMs exhibited attenuated dextran uptake both at baseline and after chlamydial stimulation (Figure 3I–K). Furthermore, *C. muridarum*-stimulated Ccr2^−/−^-BMDMs exhibited increased PI^+^ staining (Figure 3L,M) and elevated LDH release (Figure 3N), indicating severe membrane damage and cell death. Under light microscopy, Ccr2^−/−^-BMDMs exhibited noticeable swelling and large membrane bubbles at 24 h p.i., characteristic signs of pyroptosis (Figure 3O).

Collectively, these results demonstrate that CCR2 acts as a cell-intrinsic master regulator of macrophage effector functions during *C. muridarum* infection, orchestrating migration, M1 polarization, and endocytic activity while promoting cell survival. The profound impairment of antimicrobial defenses in CCR2-deficient macrophages underscores the non-redundant role of this chemokine receptor in host protective immunity against intracellular bacterial infection.

### 3.4. Ccr2 Deficiency Drives NLRP3/Caspase-3/GSDME-Mediated Pyroptosis and Enhances ROS Production in the Lung During C. muridarum Infection

**Figure 4:**
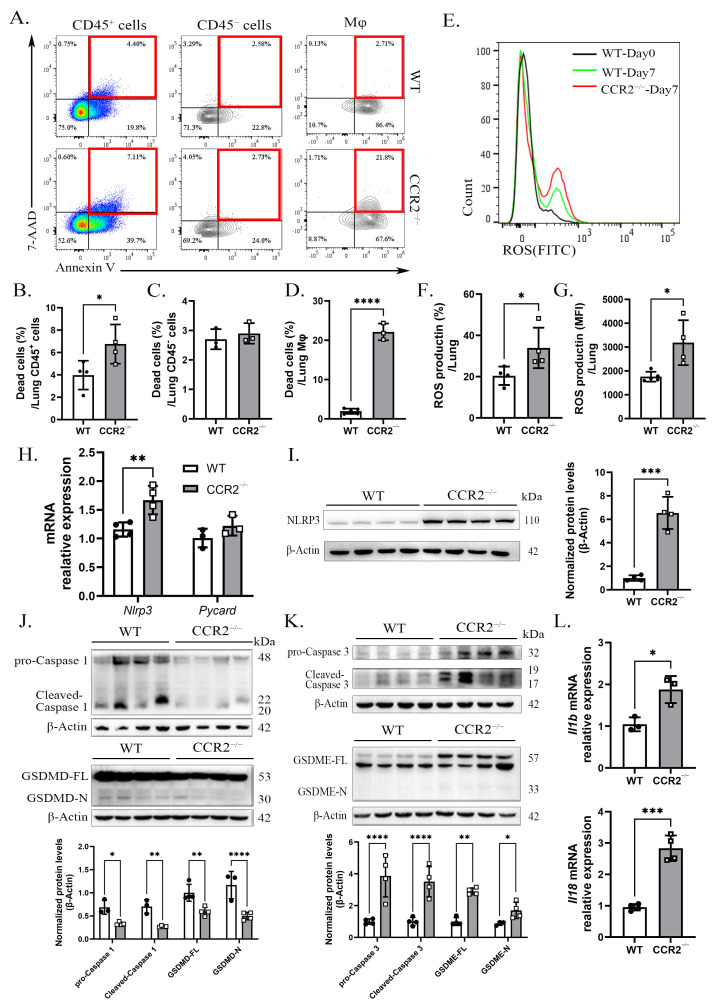
CCR2** **deficiency** **promotes** **NLRP3/Caspase-3/GSDME-mediated pyroptosis during *C. muridarum* respiratory infection. (**A**) Representative flow cytometry plots of Annexin V and 7-AAD staining in pulmonary CD45^+^ cells, CD45^−^ cells, and Macrophages (Mφ) from WT and Ccr2^−/−^ mice at day 7 p.i. (**B**–**D**) The percentage of Annexin V^+^7-AAD^+^ cells among pulmonary CD45^+^ cells (**B**), CD45^−^ cells (**C**), and Mφ (**D**). (**E**) Flow cytometry analysis of ROS production in lung cells from WT and Ccr2^−/−^ mice at day 7 p.i. Black line: naive WT mice, green line: infected WT mice, red line: infected Ccr2^−/−^ mice. (**F**,**G**) The percentage of FITC^+^ cells (**F**) and the MFI of FITC (**G**) in WT and Ccr2^−/−^ mice at day 7 p.i. (**H**) Relative mRNA expression levels of *Nlrp3* and *Pycard* were quantified by qPCR in lung tissues from WT and Ccr2^−/−^ mice at day 7 p.i., normalized to *Actb*. (**I**–**K**) Representative Western blot bands and the corresponding quantification (normalized to β-Actin) of NLRP3 (**I**), Caspase-1, GSDMD (**J**), Caspase-3, and GSDME (**K**) protein levels in lung tissues from WT and Ccr2^−/−^ mice at day 7 p.i. (**L**) Relative mRNA expression levels of *Il1b* and *Il18* were quantified by qPCR in lung tissues from WT and Ccr2^−/−^ mice at day 7 p.i., normalized to *Actb*. Data are shown as mean±SD (*n* = 3–4 mice per genotype and time point) and are representative of three independent experiments. Statistical significance was determined by unpaired Student’s *t*-test or two-way ANOVA. * *p* < 0.05, ** *p* < 0.01, *** *p* < 0.001, **** *p* < 0.0001.

We previously reported that CCR2 deficiency exacerbates pulmonary immunopathology during chlamydial respiratory infection [20]. Having shown that CCR2 deficiency impairs macrophage polarization, endocytosis, and survival both in vivo and in vitro (Figure 2 and Figure 3), we next asked which mechanism underlies this aggravated disease. At day 7 p.i., flow cytometry revealed a marked increase in Annexin V^+^7-AAD^+^ cells (defined as dead cells) among CD45^+^ leukocytes, particularly macrophages, but not in CD45^−^ parenchymal cells (Figure 4A–D). Thus, CCR2 selectively preserves immune cell viability during chlamydial infection.

Reactive oxygen species (ROS) are known drivers of cell death and inflammation [21]. Indeed, the lungs of Ccr2^−/−^ mice displayed elevated ROS levels within the CD45^+^ compartment, as shown by both the frequency of FITC^+^ cells and the MFI (Figure 4E–G). Because ROS can trigger inflammasome-dependent pyroptosis [22,23,24], we assessed the NLRP3 pathway. *Nlrp3* mRNA and protein were significantly upregulated in Ccr2^−/−^ lungs, whereas *Pycard* remained unchanged (Figure 4H,I). Unexpectedly, canonical pyroptosis effectors pro-Caspase-1, cleaved Caspase-1, GSDMD, and GSDMD-N were all suppressed (Figure 4J). This paradoxical finding suggested that an alternative pyroptotic pathway might be engaged. Recent work has identified Caspase-3 as an upstream activator of GSDME-mediated pyroptosis [25,26]. In Ccr2^−/−^ mice, we observed increased pro- and cleaved Caspase-3, elevated GSDME-N fragments (Figure 4K), and upregulated *Il1b* and *Il18* mRNA expression (Figure 4L), indicating significant activation of the Caspase-3/GSDME signaling pathway.

Collectively, these data uncover a previously unrecognized immunoregulatory function of CCR2 that limits ROS production and suppresses both the NLRP3 inflammasome and the Caspase-3/GSDME pyroptotic axis. Conversely, CCR2 deficiency unleashes this dual pyroptotic cascade, thereby contributing to the aggravated pulmonary pathology during chlamydial respiratory infection.

### 3.5. Ccr2 Deficiency Drives NLRP3-Dependent Pyroptosis via the Caspase-3/GSDME Axis in C. muridarum-Infected BMDMs

**Figure 5:**
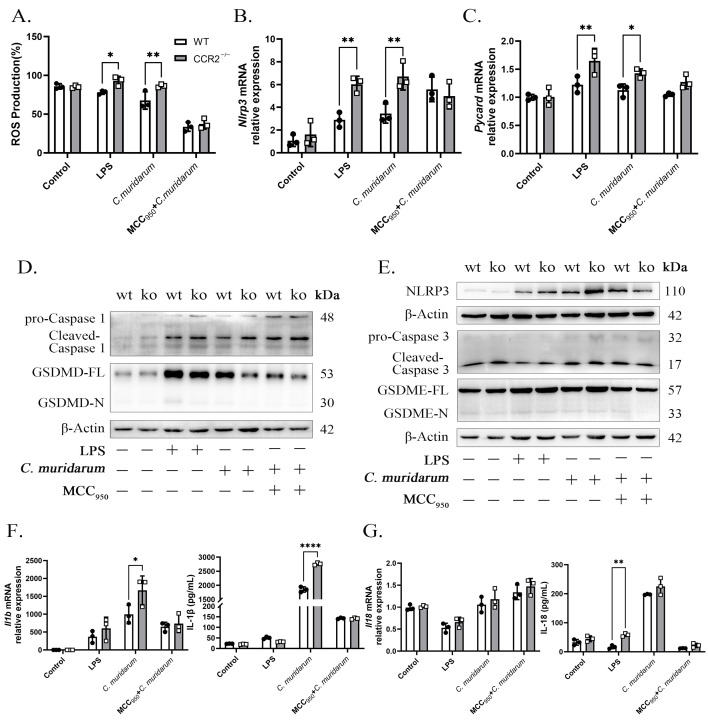
CCR2 deficiency enhanced ROS production and activated the NLRP3/Caspase-3/GSDME/IL-1β axis in *C. muridarum*-stimulated BMDMs. WT-BMDMs and Ccr2^−/−^-BMDMs were separately established as four experimental groups: Control, LPS, *C. muridarum*, and MCC_950_+*C. muridarum*. (**A**) Flow cytometry analysis of ROS production in different groups. (**B**,**C**) Relative mRNA expression levels of *Nlrp3* (**B**) and *Pycard* (**C**) were quantified by qPCR and normalized to *Actb*. (**D**) The protein expression levels of Caspase-1 and GSDMD were quantified by Western blot. (**E**) The protein expression levels of NLRP3, Caspase-3 and GSDME were quantified by Western blot. (**F**,**G**) Relative mRNA expression levels and protein secretion levels of IL-1β (**F**) and IL-18 (**G**). Data are presented as means ± SD from three independent wells per group and are representative of three independent experiments. Statistical significance was determined by two-way ANOVA. * *p* < 0.05, ** *p* < 0.01, **** *p* < 0.0001.

We previously showed that CCR2 deficiency drives NLRP3/Caspase-3/GSDME-mediated pyroptosis in the infected lung (Figure 4). To determine whether this pyroptotic pathway operates cell-intrinsically in macrophages, we established an in vitro *C. muridarum* infection model using BMDMs from WT and Ccr2^−/−^ mice. Upon stimulation with LPS and *C. muridarum*, Ccr2^−/−^-BMDMs produced significantly higher levels of ROS than WT controls as measured by DCFDA fluorescence (Figure 5A). This enhanced oxidative stress was accompanied by marked upregulation of the inflammasome-related transcripts *Nlrp3* and *Pycard* (ASC) (Figure 5B,C), indicating a heightened priming capacity in CCR2-deficient macrophages. Notably, Caspase-1 activation remained unaltered, and GSDMD activation was unexpectedly reduced (Figure 5D). This finding was consistent with our in vivo observation of suppressed canonical pyroptosis. By contrast, *C. muridarum* stimulation triggered a robust increase in NLRP3 protein, Caspase-3 activation, and GSDME expression specifically in Ccr2^−/−^-BMDMs (Figure 5E). Proteolytic cleavage of GSDME generated the active N-terminal fragment (GSDME-N), directly linking Caspase-3 to pyroptotic cell death. Cytokine profiling revealed a selective dysregulation in *Ccr2*^-/-^-BMDMs. These cells produced and secreted markedly more IL-1β (Figure 5F) but showed preserved IL-18 handling (Figure 5G). Pharmacological inhibition experiments demonstrated the central role of NLRP3 inflammasome activity in this process. Pretreatment with MCC_950_, a specific NLRP3 inhibitor, effectively normalized the exaggerated pyroptotic response in Ccr2^−/−^-BMDMs to WT-BMDMs levels (Figure 5).

Together, these data identify CCR2 as a critical cell-intrinsic brake on pyroptotic signaling in macrophages. Loss of CCR2 unleashes an NLRP3 inflammasome-dependent Caspase-3/GSDME activation cascade, and the rescue by MCC_950_ establishes NLRP3 as the primary upstream regulator of this CCR2-controlled pyroptotic axis.

### 3.6. CCR2-Deficient Macrophages Differentially Modulate CD4^+^ T Cell Responses Through Distinct Mechanisms

Our previous results showed that CCR2 deficiency enhances IL-1β production in macrophages via the NLRP3/Caspase-3/GSDME pyroptotic axis (Figure 4 and Figure 5). Given that macrophage polarization shapes Th cell immunity [27,28] and that we previously observed skewed Th2/Th17 responses with impaired Th1 immunity in Ccr2^−/−^ mice during *C. muridarum* pulmonary infection [20], we asked whether CCR2-deficient macrophages differentially regulate T cell responses through distinct interaction modes.

**Figure 6:**
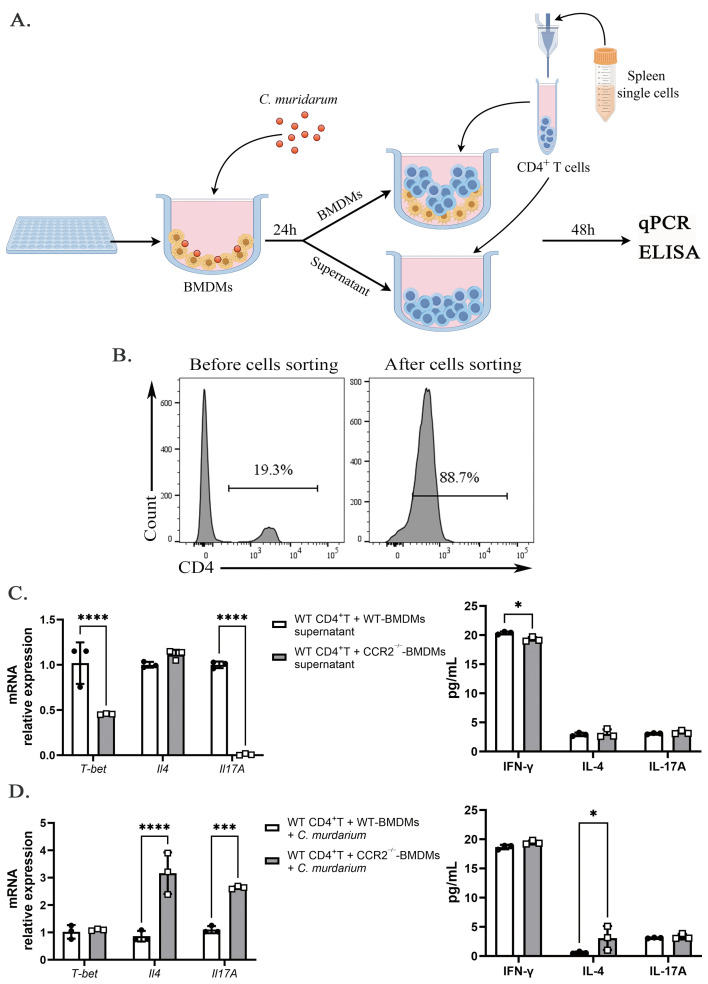
*C. muridarum*-stimulated BMDMs co-cultured with CD4^+^ T cells. (**A**) Schematic of the BMDMs-CD4^+^ T cells co-culture experimental design. (**B**) Purity of magnetic bead-sorted CD4^+^ T cells from naïve WT mouse spleens assessed by flow cytometry. (**C**) Supernatants from WT or Ccr2^−/−^-BMDMs stimulated with *C. muridarum* for 24 h were incubated with CD4^+^ T cells for 48 h. qPCR for *T-bet*, *Il4*, and *Il17a*. ELISA for IFN-γ, IL-4, and IL-17A. (**D**) WT or Ccr2^−/−^-BMDMs were stimulated with *C. muridarum* for 24 h and then co-cultured with CD4^+^ T cells for 48 h. qPCR and ELISA analyses as in (**C**). Data are presented as means ± SD from six independent wells per group and are representative of three independent experiments. Statistical significance was determined by two-way ANOVA. * *p* < 0.05, *** *p* < 0.001, **** *p* < 0.0001.

To explore this, we established an in vitro co-culture system. *C. muridarum*-stimulated BMDMs from WT or Ccr2^−/−^ mice were incubated with naïve CD4^+^ T cells (Figure 6A). Specifically, BMDMs were stimulated with *C. muridarum* for 24 h, then either co-cultured with magnetic bead-sorted CD4^+^ T cells (>88% purity, Figure 6B) at a 1:10 ratio (BMDMs to T cells) for 48 h, or the BMDM supernatants were added to T cells for 48 h. Supernatants from Ccr2^−/−^-BMDMs significantly suppressed *T-bet* mRNA expression and IFN-γ secretion in CD4^+^ T cells compared to WT-BMDM supernatants (Figure 6C). This finding indicates that factors secreted by CCR2-deficient macrophages inhibit Th1 differentiation, consistent with the attenuated pulmonary Th1 response observed in vivo. Notably, while Ccr2^−/−^-BMDMs supernatants also reduced *Il17A* mRNA expression (Figure 6C), direct cell–cell contact upregulated both *Il4* and *Il17A* transcripts as well as IL-4 secretion (Figure 6D). Thus, CCR2-deficient macrophages employ a context-dependent regulatory mechanism: soluble factors suppress Th1 and Th17, whereas direct interaction promotes Th2 and Th17 differentiation.

Collectively, these data establish CCR2 as a critical regulator of macrophage-mediated T cell polarization through two distinct mechanisms. CCR2 deficiency potentiates pyroptosis-dependent IL-1β secretion, which in turn suppresses Th1 cell development. Simultaneously, Ccr2^−/−^-BMDMs drive Th2 and Th17 differentiation via direct cellular interactions and antigen presentation. This dysregulated immunity leads to defective clearance of *C. muridarum*, driving exacerbated immunopathology and tissue damage at infection sites.

## 4. Discussion

In this study, we demonstrate that CCR2 plays a multifaceted role in host defense against *C. muridarum* respiratory infection by orchestrating monocyte trafficking, macrophage polarization, pyroptotic cell death, and subsequent T cell responses. Our findings reveal a previously unrecognized immunoregulatory axis wherein CCR2 restricts NLRP3/Caspase-3/GSDME-mediated pyroptosis and thereby prevents excessive IL-1β production, which would otherwise suppress protective Th1 immunity while allowing pathogenic Th2/Th17 responses.

CCR2 deficiency resulted in the sequestration of Ly6C^hi^ monocytes in the bone marrow and their concomitant reduction in blood, spleen, and lungs, confirming the non-redundant requirement for CCR2 in inflammatory monocyte egress. Notably, Ly6C^low^ patrolling monocytes were also diminished in peripheral blood and tissues of Ccr2^−/−^ mice, implying a broader contribution of CCR2 signaling to monocyte distribution than previously recognized. This differential outcome correlates with their divergent CCR2 profiles. Ly6C^hi^ Mo express high CCR2 and exit the marrow in a strictly CCR2-dependent manner upon inflammatory challenge [29], whereas Ly6C^low^ Mo exhibit negligible CCR2. The unexpected impact on the patrolling subset thus extends the functional purview of CCR2 beyond direct ligand-guided emigration. These data refine our understanding of monocyte trafficking hierarchies and underscore the need to examine whether CCR2 loss impinges on additional effector properties of Ly6C^hi^ Mo.

Previous studies have demonstrated that Ly6C^hi^ Mo can differentiate into proinflammatory M1-like macrophages that secrete TNF-α and IL-1β to eliminate pathogens [30,31,32,33] yet can cause tissue injury upon excessive activation [34], whereas Ly6C^low^ Mo primarily exert anti-inflammatory and tissue reparative functions by patrolling the vascular microenvironment and have been reported to differentiate into M2-like macrophages during chronic inflammation [35,36,37]. In our study, we observed that the F4/80^hi^ macrophages present in Ccr2^−/−^ lungs were predominantly associated with Ly6C^low^ monocytes rather than with Ly6C^hi^ monocytes. Whether this reflects true differentiation from Ly6C^low^ monocytes into macrophages or merely altered expression of F4/80 and CD11c markers remains to be determined. If a compensatory transition does occur, it might be sustained by CX3CR1, which is highly expressed on Ly6C^low^ monocytes and is known to support CCR2-independent migration in other infection models [38,39,40]. The reported anti-inflammatory properties of Ly6C^low^ Mo could theoretically favor their transition toward a macrophage-like phenotype, potentially contributing to pulmonary immune homeostasis [41]. However, direct experimental evidence supporting these speculations is currently lacking. Future studies using CX3CR1 blockade or knockout mice, combined with lineage-tracing approaches, will be required to directly test whether Ly6C^low^ monocytes serve as a genuine compensatory source of macrophages in the absence of CCR2.

Macrophages exhibit remarkable functional plasticity and dynamically adapt their phenotype to microenvironmental cues, thereby coordinating immune defense, inflammation, and tissue repair [42]. Maintaining a proper balance between proinflammatory M1-like and anti-inflammatory M2-like polarization is critical for immune homeostasis. In Ccr2^−/−^ mice, pulmonary macrophages displayed reduced M1-like markers and elevated M2-like markers, indicating a shift toward alternative activation. However, in vitro-stimulated *Ccr2*^-/-^-BMDMs failed to upregulate M2-like markers, establishing that the observed in vivo M2-like skewing is not cell-intrinsic but rather a compensatory adaptation to the altered inflammatory milieu caused by CCR2 deficiency. This adaptation may explain the eventual convergence of disease outcomes with wild-type mice at later stages [20]. Additional factors, such as the duration of stimulation and baseline polarization differences between BMDMs and tissue-resident macrophages, may also contribute to this discrepancy. Interestingly, when we focused solely on the LPS-stimulated groups, the marked reductions in CD86 and *Nos2* observed in Ccr2^−/−^-BMDMs under *C. muridarum* infection were not recapitulated, suggesting that CCR2 deficiency modulates macrophage polarization in a context-dependent manner. We therefore speculate that loss of CCR2 skews macrophages toward an M2-like state, thereby reducing their sensitivity to classical M1 signals. This speculation is consistent with previous studies demonstrating M2 polarization in CCR2-deficient macrophages under various inflammatory conditions, including LPS-induced ARDS [43]. Beyond polarization, we identified that CCR2-deficient macrophages possess impaired endocytic capacity both in vivo and in vitro, revealing a previously unrecognized function of CCR2 that extends beyond its canonical role in chemotaxis. Notably, the absence of an endocytic defect under steady-state conditions suggests potential compensation by tissue-resident macrophages, a hypothesis that remains to be formally tested.

Membrane integrity loss, as indicated by PI positivity and LDH release, together with typical morphological changes such as cell swelling and plasma membrane ballooning, represents the cornerstone evidence supporting pyroptotic cell death. In the present study, *C. muridarum*-stimulated Ccr2^−/−^-BMDMs exhibited significantly increased PI^+^ staining (Figure 3L,M) and elevated LDH release (Figure 3N), indicating severe membrane damage. Furthermore, under light microscopy, these cells demonstrated noticeable swelling and large membrane bubbles at 24 h p.i., characteristic signs of pyroptosis (Figure 3O). These observations are consistent with the established literature: activation of the Caspase-3/GSDME pathway induces pore formation in the plasma membrane, leading to LDH release and morphological features such as cell swelling and balloon-like bubbling [44]. Moreover, during *C. muridarum* respiratory infection, elevated levels of caspase-3 and gasdermin E proteins have been detected in infected lungs, demonstrating that Chlamydia infection does induce pyroptosis [45]. Nevertheless, while these morphological and biochemical features are highly suggestive of pyroptosis, they are not entirely specific. Necroptosis can also present with plasma membrane rupture, and late-stage apoptotic cells undergoing secondary necrosis may exhibit overlapping characteristics. A previous comparative study has demonstrated that pyroptosis undergoes membrane blebbing and produces apoptotic body-like cell protrusions prior to membrane rupture, whereas necroptosis shows an explosion-like rupture pattern [46]. Given that our evidence for the Caspase-3/GSDME axis remains indirect, functional validation, including GSDME knockdown or pharmacological inhibition, would provide definitive mechanistic evidence. Future studies employing such approaches will be necessary to conclusively establish the role of the Caspase-3/GSDME pathway in CCR2-regulated pyroptosis during intracellular bacterial infection.

Our study demonstrated that Ccr2^−/−^ mice and BMDMs following respiratory Chlamydia infection exhibited increased ROS production, elevated NLRP3 expression, and activation of the Caspase-3/GSDME pyroptotic pathway, which is consistent with the literature showing that ROS can induce NLRP3 inflammasome activation and trigger pyroptosis via Caspase-3/GSDME [47,48,49,50]. Nevertheless, we acknowledge that our current data provide correlative rather than causal evidence for the proposed ROS-NLRP3-Caspase-3/GSDME axis. Extensive studies have shown that ROS scavengers such as NAC or Mito-TEMPO effectively suppress NLRP3 inflammasome activation and downstream pyroptosis, and selective NLRP3 inhibitors like MCC_950_ directly block inflammasome assembly. In our study, MCC_950_ treatment normalized the exaggerated pyroptotic response in Ccr2^−/−^-BMDMs (Figure 5H), positioning NLRP3 as a key mediator downstream of CCR2. However, direct experiments using ROS inhibitors are needed to definitively establish whether ROS directly drives NLRP3 activation and downstream pyroptosis in our model. Future studies employing ROS scavengers or genetic approaches will be essential to confirm that CCR2 deficiency promotes pyroptosis through a ROS-dependent NLRP3 inflammasome pathway.

Excessive reactive oxygen species (ROS) drive inflammasome-dependent pyroptosis [21,22,23,24]. In this study, we found that CCR2 deficiency enhances ROS production while suppressing canonical Caspase-1/GSDMD pyroptosis and hyperactivating the Caspase-3/GSDME axis, elevating IL-1β and IL-18. How IL-1β is processed in the absence of active Caspase-1 remains unresolved, and we did not examine whether GSDME deficiency rescues the aggravated pathology in Ccr2^−/−^ mice. Future studies using Ccr2^−/−^Gsdme^-/-^ double-knockout mice and assays distinguishing mature from pro-IL-1β will address these limitations. In this study, we propose that CCR2 loss amplifies Chlamydia-induced tissue injury by augmenting GSDME-dependent pyroptosis, reflecting a compensatory host defense that dismantles the intracellular niche of the pathogen while recruiting proinflammatory effectors, despite impairing M1 polarization and endocytosis.

Pyroptosis driven by ROS enhances T cell activation via cytoplasmic damage-associated molecular patterns (DAMPs) [51], and IL-1β signaling critically regulates Th1, Th2, and Th17 differentiation [52]. In our co-culture experiments, soluble factors from Ccr2^−/−^ BMDMs suppressed Th1 and Th17 differentiation (likely due to elevated IL-1β paradoxically inhibited by IL-10 or TGF-β), while direct cell–cell contact promoted Th2 and Th17 responses via costimulatory molecules, consistent with in vivo impaired Th1 but enhanced Th2/Th17 responses during *C. muridarum* infection [20]. Future studies will employ macrophage-specific conditional knockouts to distinguish myeloid from other cell types, as well as in vivo blockade of NLRP3 or GSDME to test causality. This study demonstrates that CCR2 coordinates lung immune responses through IL-1β-dependent microenvironmental modification and macrophage-T cell interactions, limiting pathogen survival while minimizing tissue damage.

## 5. Conclusions

**Figure 7:**
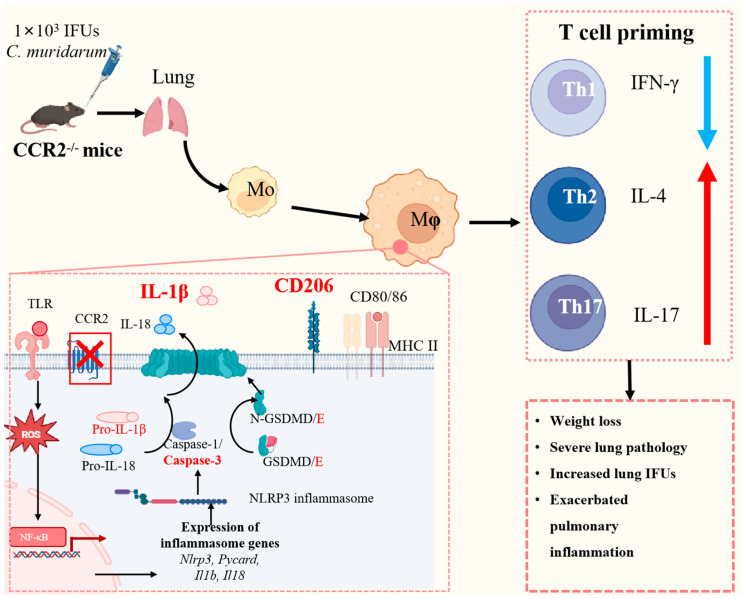
Schematic model of CCR2 enhancing anti-intracellular bacterial infection by modulating macrophage pyroptosis to rebalance Th immune responses. In this study, we demonstrate that CCR2 deficiency impairs monocyte egress from bone marrow to peripheral circulation during Chlamydia respiratory infection, resulting in reduced monocyte infiltration in infected lung tissues and skewed differentiation toward macrophages with attenuated M1 but enhanced M2 polarization. Mechanistically, CCR2 deficiency triggers excessive ROS production, upregulating the transcriptional expression of inflammasome components (NLRP3, PYCARD) and their substrates (IL-1β, IL-18), thereby promoting inflammasome priming and assembly. This process facilitates Caspase-3-mediated cleavage and activation of IL-1β and GSDME, ultimately inducing pyroptosis. The combined effects of M2-polarized macrophages and proinflammatory cytokines released during pyroptosis collectively reshape pulmonary T-cell immunity, suppressing Th1 responses while augmenting Th2-/Th17-cell polarization. Consequently, CCR2-deficient mice exhibit exacerbated disease severity upon Chlamydia infection. In the figure, the blue arrow indicates inhibition, and the red arrow indicates augmenting.

In summary, our work shows that CCR2 controls monocyte recruitment, whereas CCR2 deficiency drives monocyte-to-macrophage differentiation and pyroptosis upon infection, subsequently modulating T cell responses. We propose that CCR2 normally limits the NLRP3/Caspase-3/GSDME axis, preventing excessive IL-1β release that would otherwise skew immunity away from Th1 toward Th2/Th17. In the absence of CCR2, this brake is lost, leading to dysregulated pyroptosis, defective bacterial control, and immunopathology (Figure 7). These findings reveal a previously unappreciated role of CCR2 in integrating pyroptosis and T cell polarization, opening new directions for investigating cell-type-specific CCR2 functions and the therapeutic potential of targeting the pyroptotic cascade.

## Figures and Tables

**Table 1:** qPCR primer sequences.

Gene Name	Forward Sequence (5′-3′)	Reverse Sequence (5′-3′)
*Actb*	GGCTGTATTCCCCTCCATCG	CCAGTTGGTAACAATGCCATGT
*Nlrp3*	ATTACCCGCCCGAGAAAGG	TCGCAGCAAAGATCCACACAG
*Pycard*	CTGCTCAGAGTACAGCCAGAAC	CTGTCCTTCAGTCAGCACACTG
*Il1b*	GAAATGCCACCTTTTGACAGTG	TGGATGCTCTCATCAGGACAG
*Il18*	GACAGCCTGTGTTCGAGGATATG	TGTTCTTACAGGAGAGGGTAGAC
*Tbet*	AACCGCTTATATGTCCACCCA	CTTGTTGTTGGTGAGCTTTAGC
*Il4*	GGTCTCAACCCCCAGCTAGT	GCCGATGATCTCTCTCAAGTGAT
*Il17a*	TCAGCGTGTCCAAACACTGAG	CGCCAAGGGAGTTAAAGACTT
*Ccr2*	ATCCACGGCATACTATCAACATC	AAGGCTCACCATCATCGTAG
*Nos2*	ACATCGACCCGTCCACAGTAT	CAGAGGGGTAGGCTTGTCTC
*Mrc1*	CTCTGTTCAGCTATTGGACGC	CGGAATTTCTGGGATTCAGCTTC

## Data Availability

The original contributions presented in this study are included in the article/. Further inquiries can be directed to the corresponding author.
